# Organic Mulching Enhances Soil Health and Fungal Diversity to Promote Growth of *Aralia continentalis* Kitag: A Sustainable Alternative to Conventional Fertilization in Agroecosystems

**DOI:** 10.3390/biology14111624

**Published:** 2025-11-19

**Authors:** Qian Liu, Junyan Zheng, Yuhe Xing, Xingchi Guo, Ying Qu, Zhiyu Dong, Wei Yu, Guoyu Zhang

**Affiliations:** 1 College of Landscape Architecture, Changchun University, Changchun 130012, China; 2 Institute of Resource Utilization and Soil Conservation, Changchun University, Changchun 130022, China

**Keywords:** organic amendment, rhizosphere ecology, soil microbial network, saprotrophic fungi, nutrient cycling, medicinal plant cultivation, black soil restoration

## Abstract

Soil degradation from long-term chemical fertilization poses serious challenges to the sustainability of black soil agroecosystems in Northeast China, particularly for the cultivation of medicinal plants such as *Aralia continentalis* Kitag. To evaluate eco-friendly alternatives, we compared decomposed leaf mulching (LM), conventional fertilization (CF), and an untreated control (CK) in a five-year field experiment. LM significantly improved soil structure by reducing bulk density by 12.8% (*p* < 0.05) and increasing porosity by 15.6% while enhancing organic carbon and humus fractions by 23.4% and 31.7%, respectively. These changes promoted microbial biomass carbon by 28.2% (*p* < 0.01) and enriched beneficial fungi such as *Mortierella*, which correlated with nutrient mobilization and plant growth. Fungal richness and diversity were higher under LM (+18.4% and +12.6%, respectively), whereas CF reduced evenness and favored dominance of stress-tolerant taxa. Functional predictions indicated that LM sustained saprotrophic and symbiotic guilds, while CF weakened mycorrhizal associations. Structural equation modeling identified microbial community composition as a central mediator linking soil properties, microbial diversity, and biomass (R^2^ = 0.78), with LM exerting the strongest cascading effects. At the plant level, LM achieved the highest above- and belowground biomass, outperforming CF and CK by 26.3% and 34.5%, respectively. Overall, decomposed leaf mulching represents a sustainable strategy to restore soil quality, enhance microbial diversity, and support medicinal plant cultivation in cold-region agroecosystems.

## Simple Summary

Excessive use of chemical fertilizers has degraded black soils in Northeast China, threatening sustainable agriculture and the cultivation of medicinal plants. We tested whether organic mulching with decomposed leaf litter could improve soil health and plant growth compared with conventional fertilization and no treatment. A five-year field experiment with *Aralia continentalis* Kitag measured soil properties, microbial biomass, fungal diversity, and plant productivity. Leaf mulching improved soil structure, increased organic carbon and humus, and enriched beneficial fungi such as *Mortierella*. These changes enhanced microbial diversity and stability, leading to greater above- and belowground biomass. In contrast, chemical fertilization gave moderate yield benefits but reduced fungal diversity and community balance. Overall, decomposed leaf mulching is a low-cost, eco-friendly practice that restores soil fertility, sustains microbial functions, and supports resilient medicinal plant cultivation in cold-region agroecosystems.

## 1. Introduction

The Mollisol region of Northeast China is renowned for its exceptional fertility and agricultural productivity. However, decades of intensive cultivation and heavy reliance on chemical fertilizers have led to pronounced soil degradation [1,2]. This degradation is manifested through soil compaction, diminished organic matter, and declining biodiversity, all of which threaten the sustainability of agroecosystems and the production of regionally important medicinal crops [3,4,5]. Although chemical fertilization can provide short-term yield benefits, its continued use often accelerates soil deterioration and disturbs microbial community equilibrium, further undermining soil quality [6]. These issues underscore the urgent need for ecologically sound management practices to restore soil health and ensure the sustainable cultivation of medicinal plants.

*Aralia continentalis* Kitag, a medicinally valuable *Apiaceae* species closely related to Angelica, is endemic to northeastern China and faces significant cultivation challenges rooted in its biological and environmental requirements [7]. Its growth is constrained by regional climatic conditions and specific edaphic needs, which intersect with national conservation policies, creating a critical need for sustainable cultivation strategies [8]. Traditional approaches, such as organic amendments and subsoiling to mitigate compaction, have been employed but lack systematic optimization within the context of modern soil health and resource efficiency goals [9]. While prior studies have characterized the basic agronomic requirements of *A. continentalis*, they have largely focused on descriptive cultivation parameters rather than addressing the integrated challenges of climate adaptability and long-term yield stability [10,11]. This gap is particularly pronounced in northeastern China, where the interplay between the species’ biological constraints and regional sustainability mandates remains underexplored [12].

Organic mulching (OM), defined as the application of decomposed plant residues on the soil surface, has been widely recognized as an effective practice to modify soil structure, increase nutrient availability, and stimulate microbial activity [13]. Although its benefits are well documented in temperate and tropical agroecosystems, its ecological role in cold regions remains insufficiently understood, particularly with regard to soil–microbe–plant interactions in medicinal plant systems [14]. Previous studies suggest that OM can reshape fungal communities involved in nutrient cycling, yet its influence on functional groups critical for plant growth under cold climatic conditions has not been comprehensively investigated [15]. This knowledge gap limits the design of soil management strategies adapted to cold agroecosystems.

This study addresses the primary research question: Can five consecutive years of leaf mulching—without chemical fertiliser—sustainably improve rhizosphere soil properties, fungal diversity, and the yield of *Aralia continentalis* Kitag in cold black-soil regions? By investigating this question, this study aims to provide valuable insights into the long-term sustainability of organic mulching in medicinal plant cultivation in Northeast China. Specifically, we hypothesize that decomposed leaf mulch enhances *Aralia continentalis* Kitag growth by improving soil physical properties, increasing nutrient availability, and promoting fungal diversity. Specifically, we propose that (1) leaf mulching reduces soil bulk density and increases porosity, facilitating root development; (2) enhanced organic carbon and nutrient availability stimulate microbial activity, accelerating nutrient mineralization and uptake by plant roots; and (3) shifts in fungal community composition, particularly the enrichment of functional groups (e.g., Ascomycota), promote plant growth by suppressing pathogens and accelerating organic matter decomposition.

To address the limitations of chemical fertilization and continuous cropping in *Aralia continentalis* Kitag cultivation, this study aimed to evaluate whether decomposed leaf mulching could enhance soil quality and plant performance by improved soil structure, nutrient availability, and fungal diversity. We compared leaf mulching (LM), conventional fertilization (CF), and an untreated control (CK) in a five-year field experiment, integrating physicochemical, microbiological, and plant growth assessments to elucidate the mechanisms linking soil improvement and microbial regulation in cold-region *Mollisols*. This long-term data provides valuable insights into the sustained effects of organic mulching, allowing us to capture the cumulative impact of mulching on soil properties, microbial communities, and plant growth, unlike most studies that focus on single-season snapshots.

## 2. Materials and Methods

### 2.1. Study Site Description

The field experiment was conducted at the Experimental Teaching Base of Changchun University in northeastern China (43°49′50″ N, 125°17′58″ E). This region experiences a temperate monsoon climate characterized by cold, dry winters and warm, humid summers, with an average annual temperature of 7.3 °C and an average annual precipitation of 860.3 mm, most of which is concentrated between June and August. The soil at the experimental site belongs to the black soil belt of Northeast China and is classified as Chernozem under the FAO/WRB system (commonly mapped as Gleyic Chernozem in the Changchun area) and as a Mollisol under the USDA Soil Taxonomy (with regional reports of Borolls, e.g., Calciboroll, in croplands around Changchun) [16].

To evaluate the effects of organic mulching on soil quality and plant growth, a field trial of *Aralia continentalis* Kitag was established in 2018 following seedling cultivation in 2017. The experiment adopted a randomized complete block design (RCBD) with three treatments: control (CK), conventional fertilization (CF), and leaf mulching (LM), with three replicates per treatment. Each plot measured 2 m × 2 m and contained 20 uniformly sized *A*. *continentalis* plants. Treatments were randomly assigned within each block using a random number generator to minimize spatial bias. Both mulch and fertilizer treatments were reapplied annually—leaf mulch in autumn after harvest and fertilizer in early spring before transplanting—to maintain treatment consistency throughout the five-year period. The field was managed under uniform agronomic practices, including irrigation and manual weeding each year. CK (Control) plots served as the baseline reference, providing a comparison against which the effects of mulching and fertilization were assessed.

### 2.2. Experimental Design

The experiment followed a randomized complete block design consisting of three treatments with three replicates each for a total of nine plots. Each plot measured 2 m × 2 m and was separated by 0.5 m buffer zones to prevent cross-contamination among treatments. The treatments were as follows: CK (Control): No mulch applied and no fertilizer provided; CF (Conventional Fertilization): Application of 150 kg/ha of synthetic fertilizers (N-P_2_O_5_-K_2_O_2_ ratio: 15-15-15) applied as a single basal dose before transplanting; LM (Leaf Mulching): Locally sourced broad-leaved forest litter (primarily Populus and *Betula* spp.) with a C:N ratio of 25–30 and ~60% decomposition level was applied at 10 t/ha (dry basis), distributed evenly to form a 3–5 cm surface layer (4 kg per plot). The average nutrient content of the mulch was 0.8% N, 0.15% P, and 0.5% K. Mulch was applied annually in autumn after harvest. It should be noted that nutrient inputs between LM and CF were not standardized to equal N-P-K rates. This design reflects real-world field management practices, where leaf mulching functions as a resource-recycling strategy providing organic matter, carbon substrates, and microbial habitat, rather than a nutrient-equivalent fertilization input. Therefore, this comparison evaluates holistic agroecological effects rather than equal-nutrient substitution. Uniform *Aralia continentalis* seedlings were transplanted at 20 plants per plot. All treatments received identical agronomic management, including manual weeding and rainfed cultivation. Soil moisture was monitored to assess mulching-induced water regulation effects. After five consecutive years of consistent management, soil and plant samples were collected during peak growth (July–August 2024) to capture cumulative treatment effects.

### 2.3. Soil and Plant Measurements

#### 2.3.1. Soil Properties

Soil samples were collected from two depths (0–10 cm and 10–20 cm) at three random points within each plot using a soil auger with a 10 cm diameter. These samples were then homogenized to obtain a composite sample. Additionally, rhizosphere soils were collected and stored at –80 °C for further analysis. The following soil parameters were determined: Bulk density was measured using the core method with a 100 cm^3^ stainless steel cylinder. Soil moisture content was determined by oven-drying the samples at 105 °C to a constant weight. pH was measured in a 1:2.5 soil-to-water suspension using a pH meter [17]. Organic carbon content was determined via dichromate oxidation using the Walkley-Black method [18]. Available nitrogen was measured using the alkaline hydrolysis diffusion method, and available phosphorus was extracted with NaHCO_3_ and analyzed using the molybdenum blue method. Available potassium was determined via ammonium acetate extraction followed by flame photometry [19]. Soil microbial biomass carbon (MBC) and microbial biomass nitrogen (MBN) were determined using the chloroform fumigation-K2SO_4_ extraction method, while microbial biomass phosphorus (MBP) was measured using the chloroform fumigation-NaHCO_3_ extraction method [20].

#### 2.3.2. Fungal Diversity Analysis

Fungal diversity was assessed using high-throughput sequencing, which was per-formed by Nanchang Kechang Bio Co., Ltd. (Nanjing, China) on an Illumina NovaSeq 6000 platform. DNA from soil samples was extracted using the Magi kit (Catalog No.: D3142-02), and the ITS1 region was amplified by PCR with primers ITS1F/ITS2R. Low-quality reads were filtered, and chimeras were removed. Operational taxonomic units (OTUs) were clustered at a 97% similarity threshold using the QIIME2 (version 2022.8) bioinformatics pipeline, and taxonomic assignments were made against the UNITE database. In addition to taxonomic classification, the FUNGuild (version 1.0) tool was employed to predict fungal functional guilds, categorizing them into groups such as saprotrophic, symbiotic, and pathogenic fungi. This allowed us to assess whether leaf mulching (LM) enriched beneficial fungal taxa, such as mycorrhizal fungi, or promoted the proliferation of pathogenic fungi. Sequencing and bioinformatics analysis were performed by Biomarker Technologies Co., Ltd. (Beijing, China).

#### 2.3.3. Plant Growth and Health Assessment

To evaluate plant responses to treatments, the following parameters were recorded at the flowering stage: Aboveground and Belowground Biomass (g/plant): Fresh and dry weight measurements after oven-drying at 65 °C [21]. Pest and Disease Incidence (%): Calculated as the proportion of affected plants per plot.

### 2.4. Integrated Metrics and Causal Insight

In this study, we used Structural Equation Modeling (SEM) to quantify the causal pathway: mulch → soil → microbes → growth. By integrating multiple soil, microbial, and plant metrics, we assessed the multifaceted impacts of organic mulching on soil health and plant growth.

### 2.5. Statistical Analysis

Data were analyzed using one-way analysis of variance (ANOVA) to compare the effects of the three treatments (CK, CF, LM) on soil properties, fungal diversity, and plant growth. Confidence intervals (CIs) at a 95% level were calculated for each treatment to assess the precision and variability in the measurements. Tukey’s HSD test was used for post hoc comparisons between treatment means (*p* < 0.05). All statistical analyses were performed using SPSS 26.0 (IBM Corp., Armonk, NY, USA). Structural equation modeling (SEM) was performed using AMOS 24.0 (IBM Corp.), and model fit was evaluated by χ^2^/df, RMSEA, CFI, and GFI indices. Principal coordinate analysis (PCoA) based on Bray–Curtis’s dissimilarity of OTU-level fungal community composition was conducted in R v.4.2.2 (vegan package). Figures were generated using OriginPro 2023 (OriginLab, Northampton, MA, USA) and R (version 4.2.2) with the ggplot2 package). The results are presented as mean ± standard error (SE), and significance was determined at *p* < 0.05.

## 3. Results and Discussion

### 3.1. Soil Physicochemical Characteristics Under Different Treatments

#### 3.1.1. Soil Physical Properties (pH, EC, Bulk Density, Porosity, Moisture)

This study analyzed the basic soil properties of the 0–10 cm and 10–20 cm soil layers during the cultivation of AC under three management practices: CK (control), LM (5-year decomposed leaf mulch), and CF (conventional fertilization). The results are summarized in Table 1.

In CK and CF, pH slightly decreased with depth, while LM maintained stable values between layers. Overall, LM showed significantly lower pH (*p* < 0.05) than CK and CF, likely due to organic acid release and humic accumulation during litter decomposition [22,23]. The mildly acidic environment under LM may have favored fungal proliferation and nutrient mobilization [24,25].

Soil moisture declined with depth but remained highest under LM, confirming its role in reducing evaporation and improving water availability [26,27]. Although lower moisture in subsoils may indicate compaction, soil penetration resistance (PR) was not measured, and this limitation should be addressed in future studies. Collectively, LM improved soil structure, water balance, and the microbial habitat, forming the foundation for enhanced nutrient cycling and plant–microbe interactions [28].

After five years of management, leaf mulching (LM) markedly improved soil structure compared with conventional fertilization (CF) and the control (CK). LM reduced bulk density by 12–15% (*p* < 0.05) and increased porosity by 18–22%, enhancing aeration and water retention. These changes reflect organic input–driven microbial aggregation and humus formation, which stabilized the soil matrix and mitigated compaction [29,30].

#### 3.1.2. Soil Chemical and Nutrient Characteristics (SOC, TN, TP, TK, Available Nutrients, Microbial Biomass Nutrients)

##### Soil Organic Carbon (SOC)

**Figure 1:**
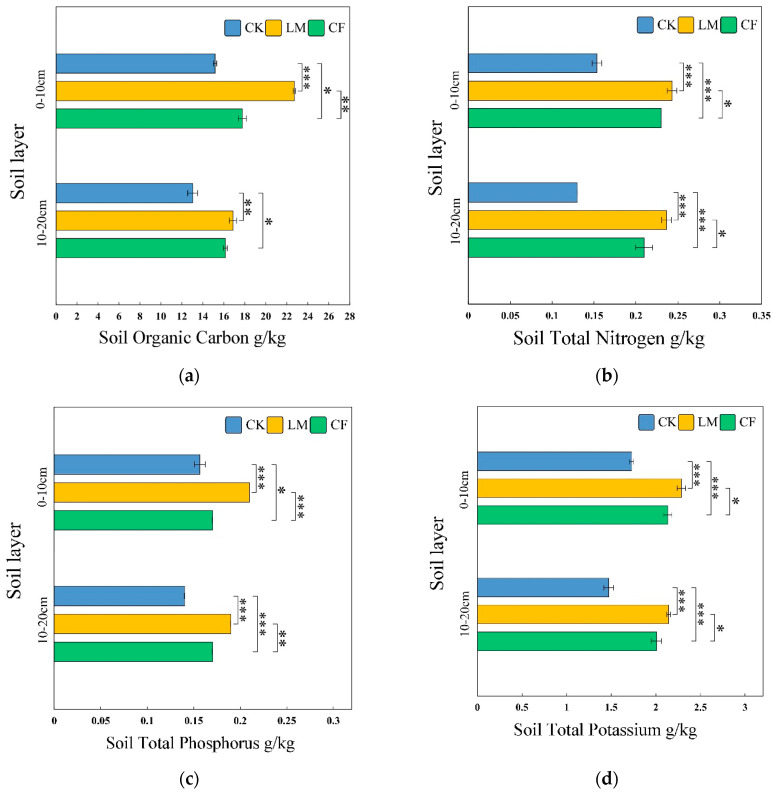
Soil organic carbon (**a**), total nitrogen (**b**), phosphorus (**c**), and potassium (**d**) in the 0–10 cm and 10–20 cm soil layers under different treatments. Error bars represent standard error (*n* = 3); *, **, and *** indicate significant differences at *p* < 0.05, *p* < 0.01, and *p* < 0.001, respectively. LM refers to leaf mulching, CF to conventional fertilization, and CK to the untreated control.

SOC is a key indicator of soil quality and carbon dynamics under different management practices [31]. Figure 1a shows that LM significantly increased SOC, especially in the topsoil (0–10 cm). LM raised SOC by 49.8% compared to CK and 29.9% compared to CF. CF increased SOC by 17.2% relative to CK, but its effect was weaker than LM. The increase in SOC under LM is likely due to sustained organic inputs and enhanced microbial metabolism [32,33]. In the subsoil (10–20 cm), LM also maintained higher SOC than CK and CF, suggesting long-term mulching improves carbon sequestration at depth. CF outperformed CK in the subsoil (+23.7%) likely due to fertilizer effects [34].

##### Soil Total Nitrogen (TN)

As shown in Figure 1b, TN concentrations were significantly influenced by management practices. In the 0–10 cm layer, LM had the highest TN content (0.24 g/kg), 60% higher than CK and 4% higher than CF. This suggests that leaf mulch enhances nitrogen accumulation, likely by stimulating microbial activity [35]. In the 10–20 cm layer, LM also outperformed CK and CF, indicating long-term mulching improves nitrogen content at depth. CF showed intermediate values, indicating that chemical fertilization increases nitrogen but with less consistency than LM [36]. These results emphasize LM’s role in maintaining soil nitrogen fertility across layers [37].

##### Soil Total Phosphorus (TP)

Figure 1c shows that LM significantly increased TP content, particularly in the 0–10 cm layer. TP under LM was 33.8% higher than CK and 23.5% higher than CF, likely due to the decomposition of organic matter in the mulch, releasing phosphorus [38]. In the 10–20 cm layer, LM still maintained higher TP than CK, although the advantage was smaller. CF elevated TP compared to CK (+21.4%) but remained lower than LM, suggesting that soluble fertilizer-derived phosphorus may leach into subsurface layers [39,40]. LM demonstrated greater stability in phosphorus content across both layers, indicating its potential for improving phosphorus retention compared to conventional fertilization.

##### Available Potassium (AK)

Figure 1d shows that LM significantly enhanced TK, particularly in the 0–10 cm layer. LM increased TK by 32.4% compared to CK and 23.1% compared to CF. This enrichment likely resulted from the mineralization of potassium-rich organic residues in the mulch [41]. In the 10–20 cm layer, LM maintained slightly higher TK than CF, but the difference was not statistically significant. The limited vertical mobility of potassium may explain the reduced effects in deeper soil [42]. LM exhibited exceptional consistency across replicates, suggesting stable long-term potassium availability, whereas CF showed greater variability, likely due to uneven distribution or leaching [43].

##### Available Nitrogen (AN)

**Figure 2:**
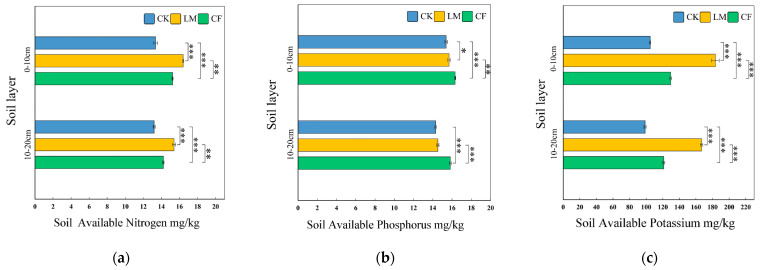
Soil Available Nitrogen (**a**), Phosphorus (**b**), and Potassium (**c**) in 0–10 cm and 10–20 cm layers under different treatments. Error bars represent SE (*n* = 3); *, **and *** denote significance at *p* < 0.05, *p* < 0.01 and *p* < 0.001, respectively. LM refers to leaf mulching, CF to conventional fertilization, and CK to the untreated control.

Figure 2a shows that LM significantly increased available nitrogen (AN) compared to CK and CF in both soil layers. In the 0–10 cm layer, LM had the highest AN (16.42 mg/kg), 22.9% higher than CK (13.36 mg/kg) and 7.7% higher than CF (15.24 mg/kg) [44]. The increase in AN under LM is likely due to the decomposition of organic residues and enhanced microbial mineralization. In the 10–20 cm layer, LM also had the highest AN (15.40 mg/kg), surpassing CF (14.21 mg/kg) and CK (13.20 mg/kg), indicating that LM improves nitrogen availability not only in surface soils but also in deeper layers [45].

##### Available Phosphorus (AP)

As shown in Figure 2b, CF led to the highest available phosphorus (AP) content in both soil layers. In the 0–10 cm layer, CF (16.32 mg/kg) exceeded CK and LM by 6.1% and 4.1%, respectively, indicating that fertilizer-derived phosphorus accumulates readily. LM showed more gradual improvements, with increases of 1.9% in surface soil and 1.6% in subsoil compared to CK [46]. This suggests that LM provides a slower but consistent release of phosphorus from organic matter decomposition, whereas CF demonstrated rapid and pronounced increases in AP [47].

##### Available Potassium (AK)

Figure 2c shows that LM significantly enhanced available potassium (AK), particularly in the 0–10 cm layer, where it was 75% higher than CK and 41% higher than CF [48]. This effect is likely due to the release of potassium from potassium-rich leaf litter. In the 10–20 cm layer, LM maintained its advantage (184 mg/kg) over both CK (109 mg/kg) and CF (134 mg/kg), although the differences were smaller at depth. The limited mobility of organic-derived potassium may explain the reduced effect in deeper soil [49,50].

##### Soil Microbial Biomass Nitrogen (SMBN)

**Figure 3:**
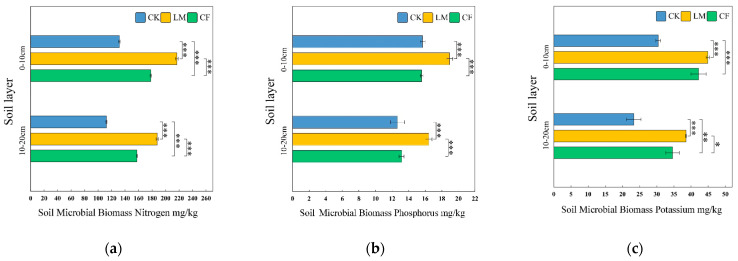
Soil Microbial Biomass Nitrogen (**a**), Phosphorus (**b**), and Potassium (**c**) in 0–10 cm and 10–20 cm layers under different treatments. Error bars represent SE (*n* = 3); *, ** and *** denote significance at *p* < 0.05, *p* < 0.01 and *p* < 0.001, respectively. LM refers to leaf mulching, CF to conventional fertilization, and CK to the untreated control.

As illustrated in Figure 3a, SMBN was significantly higher under LM compared with CK and CF at both soil depths. In the 0–10 cm layer, LM enhanced SMBN by 64.7% over CK and 21.8% over CF, confirming the strong positive effect of decomposed leaf mulching on surface microbial nitrogen [51]. In the 10–20 cm layer, LM maintained its advantage, with increases of 66.8% relative to CK and 18.9% relative to CF. Although CF also elevated SMBN compared with CK, its effect was more moderate, reaching 82.1% and 84.2% of LM levels in the topsoil and subsoil, respectively. Notably, SMBN values declined with depth across all treatments, and LM showed a 13.6% reduction between layers, reflecting the preferential enrichment of microbial nitrogen in organic-rich surface soils [52]. Overall, LM substantially enhanced microbial nitrogen pools, highlighting its sustained contribution to soil fertility.

##### Soil Microbial Biomass Phosphorus (SMBP)

As shown in Figure 3b, LM significantly increased SMBP compared with both CK and CF across depths. In the 0–10 cm layer, LM improved SMBP by 21.4% and 21.7% relative to CK and CF, respectively. Interestingly, CF and CK exhibited nearly identical values (15.57 and 15.67 mg/kg), indicating negligible P stimulation from chemical fertilization at the surface. In the 10–20 cm layer, LM still demonstrated a marked advantage, surpassing CK by 30.0% and CF by 24.9%. The slight improvement in CF over CK in the subsurface suggests limited downward mobility of fertilizer-derived phosphorus, consistent with its rapid but short-lived availability [53]. By contrast, organic inputs from LM may promote sustained microbial P through enhanced enzymatic activity (e.g., phosphatase secretion), reduced P fixation via organic chelation of Fe^3+^ and Al^3+^, and improved soil structure that mitigates leaching [54,55].

##### Soil Microbial Biomass Potassium (SMBK)

As depicted in Figure 3c, LM also exerted the strongest effect on SMBK. In the 0–10 cm layer, LM reached 44.85 mg/kg, exceeding CK (30.45 mg/kg) by 47.2% and CF (42.17 mg/kg) by 6.4%. With increasing depth, SMBK decreased under all treatments, though the rate of decline varied: CK dropped by 23.6%, CF by 17.9%, and LM by 14.1%. These results indicate that LM not only enhanced potassium in surface soils but also provided measurable benefits in subsoils, likely via gradual residue decomposition and downward nutrient infiltration [56]. Furthermore, LM displayed exceptional consistency, with minimal variation among replicates, underscoring its stable contribution to microbial potassium pools. By contrast, CF showed weaker performance at depth, while CK consistently exhibited the lowest SMBK values, reflecting nutrient limitation under natural conditions [57].

### 3.2. Soil Humus Composition and Transformation

Different management practices had significant effects on soil humus carbon, humic acid carbon, and the humic acid to fulvic acid ratio (Table 2).

#### 3.2.1. Humus Carbon

LM treatment significantly increased humus carbon concentrations compared with both CK and CF across soil layers. In the 0–10 cm layer, LM reached 10.53 g/kg, representing increases of 47.5% over CK (7.14 g/kg) and 31.0% over CF (8.04 g/kg). Although all treatments exhibited a decline in humus carbon with depth, the reduction was least pronounced under LM (9.8%) and greatest under CF (12.2%). This suggests that organic mulching not only enhanced surface carbon sequestration but also promoted downward migration of stable carbon, improving subsoil fertility more effectively than chemical fertilization. The continuous addition of high C: N organic residues in LM likely facilitated the accumulation of recalcitrant carbon pools [58].

#### 3.2.2. Fulvic Acid Carbon

Fulvic acid carbon showed distinct treatment effects between soil layers. In the topsoil, CK exhibited the highest value (3.31 g/kg), suggesting relatively strong retention of native labile organic matter. However, in the 10–20 cm layer, LM outperformed both CK and CF, reaching 2.82 g/kg compared with 2.73 g/kg in CK and 2.08 g/kg in CF. This indicates that LM promoted deeper transport and stabilization of fulvic substances, while CF failed to maintain comparable levels. The inferior performance of CF may be linked to acidification and the absence of mobile organic inputs, which limit fulvic acid accumulation in subsoil horizons [59].

#### 3.2.3. Humic Acid Carbon

LM treatment also yielded the highest humic acid carbon at both depths, particularly in the 0–10 cm layer (6.36 g/kg). This value was nearly fourfold greater than CK (1.51 g/kg) and 47% higher than CF (4.32 g/kg). Even in the subsoil, LM maintained the largest pool (5.65 g/kg), reflecting strong enhancement in humification. The superior performance of LM suggests that microbial activity stimulated by organic inputs accelerated the conversion of litter-derived carbon into stable humic substances [60]. By contrast, CF contributed moderate increases but remained inferior to LM, while CK consistently had the lowest values.

#### 3.2.4. HA/FA Ratio

The HA/FA ratio reflects the degree of humification and stability of organic matter. In the 0–10 cm layer, CF exhibited the highest ratio (1.91), followed by LM (2.06) and CK (0.46). This suggests that fertilizer inputs accelerated humification, generating more humic than fulvic acids. However, in the 10–20 cm layer, LM surpassed CF (2.01 vs. 1.74), indicating more stable and sustained humification at depth. The relatively low ratio under CF in subsoil may reflect nutrient leaching and limited downward transport of humic substances. In contrast, the continuous supply of decomposed residues in LM provided a balanced source of precursors for humus formation, thereby improving the long-term stability of soil organic matter [61].

### 3.3. Soil Microbial Characteristics Under Different Treatments

#### 3.3.1. Soil Fungal Diversity Under Different Management Practices

High-throughput sequencing demonstrated that both LM and CF treatments substantially enhanced fungal richness and diversity relative to CK (Table 3). The number of observed OTUs nearly doubled in LM (428) and CF (430) compared with CK (228), and similar increases were observed for the ACE and Chao1 richness estimators. Diversity indices further confirmed this trend: the Shannon index was higher in LM (4.40) and CF (4.38) than in CK (3.93), whereas the Simpson index was markedly lower (0.03 for LM and CF vs. 0.07 for CK), reflecting greater evenness in community composition. Coverage values exceeded 0.996 across all treatments, confirming sufficient sequencing depth and reliable taxonomic representation. Collectively, these results indicate that both organic mulching and chemical fertilization fostered richer and more diverse fungal communities, with LM showing a slight advantage in promoting balanced community structure.

#### 3.3.2. Community Composition at the Soil Phylum Level and Genus Level

**Figure 4:**
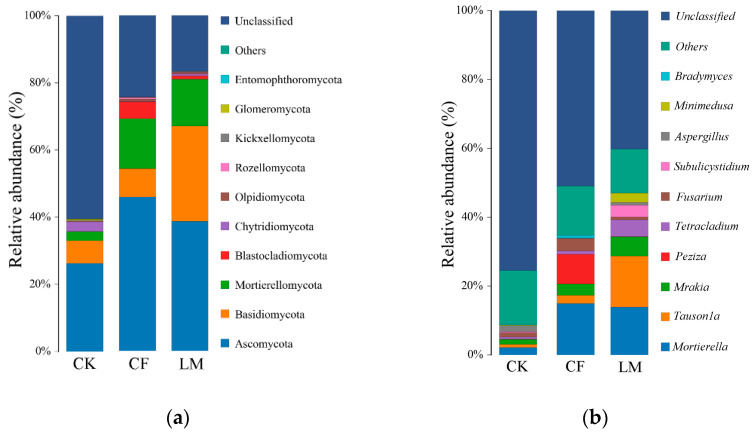
Relative abundance of soil fungal communities at the phylum (**a**) and genus (**b**) levels. LM refers to leaf mulching, CF to conventional fertilization, and CK to the untreated control.

At the phylum level (Figure 4a), pronounced differences in fungal community composition were observed among treatments. In the control (CK), Ascomycota dominated, accounting for nearly 80% of the total fungal population, with a considerable fraction of sequences remaining unclassified. Under conventional fertilization (CF), the relative abundance of Ascomycota decreased markedly, accompanied by an enrichment of Basidiomycota and Glomeromycota, along with greater representation of minor phyla. Litter mulching (LM) exhibited a similar but more pronounced shift, showing a further reduction in Ascomycota, a substantial increase in Basidiomycota, and higher proportions of Kickxellomycota and Rozellomycota compared with CK. These results suggest that both CF and LM promoted a more taxonomically diverse community by favoring fungal groups associated with organic matter turnover and symbiotic interactions.

At the genus level (Figure 4b), CK was dominated by unclassified taxa, reflecting low taxonomic resolution under nutrient-limited conditions. By contrast, CF and LM treatments significantly reshaped community composition, reducing the proportion of unclassified sequences and enriching well-defined genera such as *Mortierella*, *Tausonia*, and *Mrakia*. LM, in particular, strongly promoted *Mortierella*, a saprotrophic genus involved in organic matter decomposition, highlighting the role of organic mulching in stimulating beneficial decomposers. However, both CF and LM also increased the relative abundance of potential plant pathogenic genera such as *Fusarium*, suggesting possible ecological trade-offs between enhanced nutrient cycling and elevated pathogen risk. These shifts demonstrate that external nutrient inputs-either chemical or organic-profoundly restructure fungal communities at multiple taxonomic levels [62].

**Figure 5:**
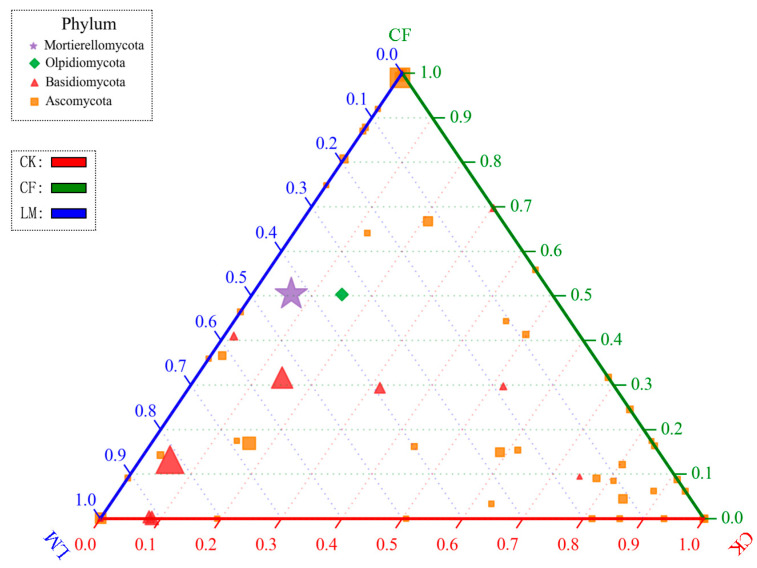
Ternary plot of dominant fungal phyla among different treatments. LM refers to leaf mulching, CF to conventional fertilization, and CK to the untreated control.

The ternary plot (Figure 5) further illustrates treatment-specific clustering of dominant fungal phyla. CK samples cluster near the Ascomycota apex, CF near Basidiomycota, while LM points are more dispersed. CK communities were primarily associated with Ascomycota, while CF-enriched soils clustered toward Basidiomycota dominance. LM samples, however, showed a broader distribution, with notable contributions from Chytridiomycota and Mortierellomycota, reflecting enhanced heterogeneity and functional diversity. These patterns suggest that fertilization and litter mulching generate distinct ecological niches, selectively favoring different fungal lineages [63,64]. Overall, the results indicate that management practices exert strong influence on soil fungal community assembly, with LM fostering a more functionally diverse and ecologically balanced fungal consortium compared to CF.

#### 3.3.3. Functional Prediction of Fungal Communities

**Figure 6:**
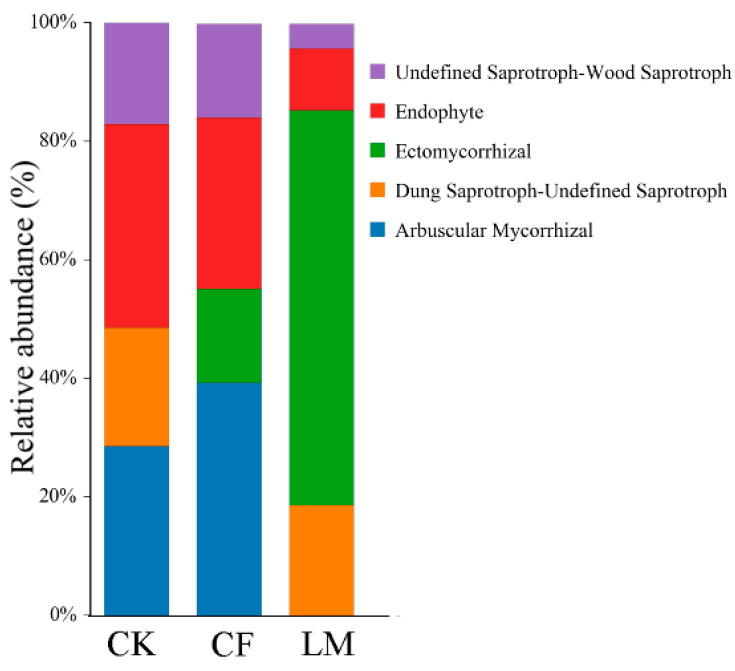
Predicted functional guild composition of fungal communities. LM refers to leaf mulching, CF to conventional fertilization, and CK to the untreated control.

Functional guild analysis revealed clear differences in trophic modes among treatments (Figure 6). In CK soils, fungal communities were almost exclusively dominated by saprotrophs, particularly undefined wood saprotrophs, reflecting the reliance of natural systems on decomposers for organic matter turnover. Under LM, saprotrophs remained the dominant guild (≈80%), indicating that organic amendments effectively sustained decomposition capacity. By contrast, CF substantially reduced saprotrophic fungi to ~60%, suggesting that chemical fertilization diminishes the ecological role of saprotrophs by altering resource availability.

Mycorrhizal fungi were strongly suppressed under fertilization. Ectomycorrhizal fungi exhibited a declining pattern (CK > LM > CF), while arbuscular mycorrhizal fungi (AMF) were detected only in CK soils. These patterns suggest that fertilization—likely through elevated phosphorus availability—interferes with plant–fungus symbioses, reducing the ecological importance of mycorrhizal associations [65].

Other guilds, including endophytes and dung saprotrophs, also followed a decreasing gradient from CK to LM to CF. Notably, dung saprotrophs completely disappeared under CF, highlighting their high sensitivity to chemical inputs [66].

Taken together, these results indicate that conventional fertilization disrupts critical saprotrophic and symbiotic guilds, thereby narrowing the functional diversity of fungal communities. In contrast, organic mulching preserved a broader spectrum of functional groups, supporting both decomposition and plant-associated symbioses. This underscores the ecological advantage of organic amendments in maintaining functionally resilient soil microbiomes. Specifically, LM enriched beneficial guilds, particularly mycorrhizal fungi, while CF led to the decline in both beneficial and saprotrophic guilds, suggesting that organic mulching supports a more diverse and functionally resilient soil microbiome compared to conventional fertilization.

### 3.4. Effects of Different Treatments on Plant Biomass Accumulation

**Figure 7:**
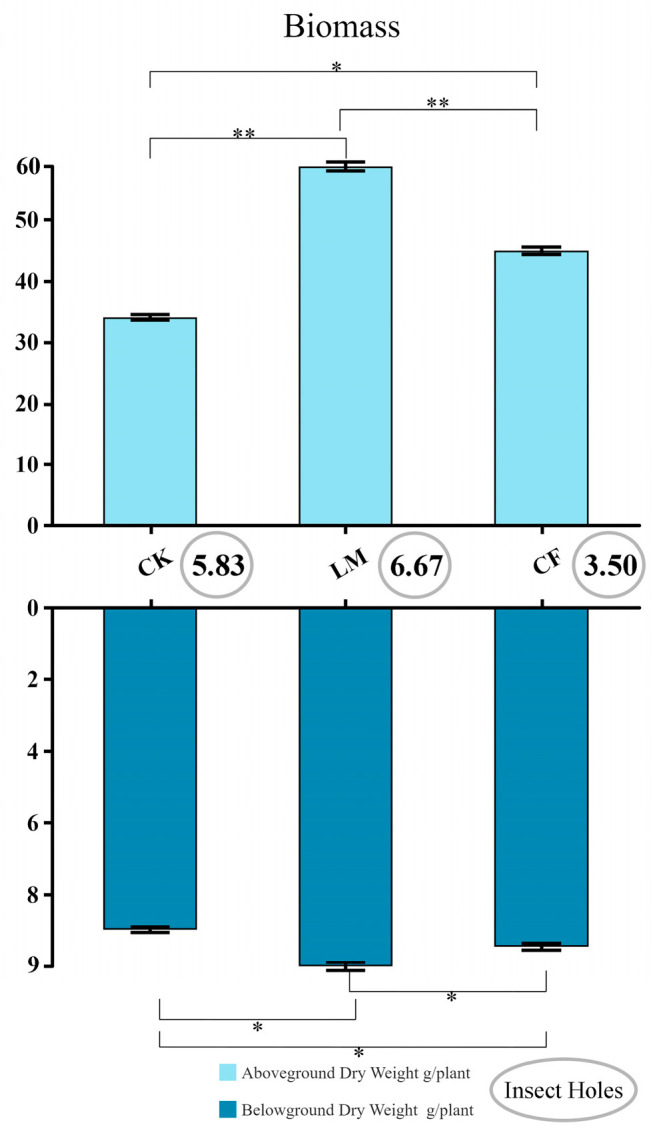
Comparison of aboveground and belowground biomass per plant, and number of insect holes. * and ** denote significance at *p* < 0.05 and *p* < 0.01, respectively. LM refers to leaf mulching, CF to conventional fertilization, and CK to the untreated control.

The comparative analysis of biomass indices revealed that management practices exerted pronounced effects on both aboveground and belowground productivity (Figure 7). Among treatments, LM consistently produced the highest biomass, confirming the beneficial role of decomposed leaf mulching in enhancing plant growth [67].

Aboveground biomass exhibited the most substantial increase under LM, significantly surpassing both CF and CK. This suggests that improvements in soil nutrient availability, moisture retention, and microbial activity under organic amendments effectively supported shoot development and canopy expansion.

Belowground biomass followed a similar, though less pronounced, trend. Root dry weights were greatest under LM, intermediate under CF, and lowest under CK. The enhanced root biomass in LM plots indicates that improved rhizosphere conditions stimulated assimilate allocation belowground. However, the relatively smaller difference compared to aboveground biomass implies a preferential investment toward shoot growth under optimal soil conditions, likely to maximize light interception and photosynthetic efficiency [68,69,70]. 

Interestingly, pest pressure, assessed through insect hole counts, was lowest under CF, whereas LM and CK showed higher incidences of leaf damage. This may be attributed to fertilizer-induced changes in plant nutritional profiles or indirect soil–plant–insect interactions [70]. Nevertheless, the ecological risks of long-term chemical fertilization, such as nutrient imbalance and soil degradation, should not be overlooked [71].

Overall, the results highlight that five years of decomposed litter mulching markedly enhanced both above- and belowground biomass accumulation, primarily through synergistic improvements in soil structure, microbial activity, and nutrient cycling. While CF provided moderate productivity gains, its effects were inferior to LM and carried potential ecological trade-offs, underscoring the advantages of organic amendments for sustainable biomass production [72].

### 3.5. Relationships Among Soil Health, Microbial Diversity, and Plant Growth

#### 3.5.1. CK Treatment

**Figure 8:**
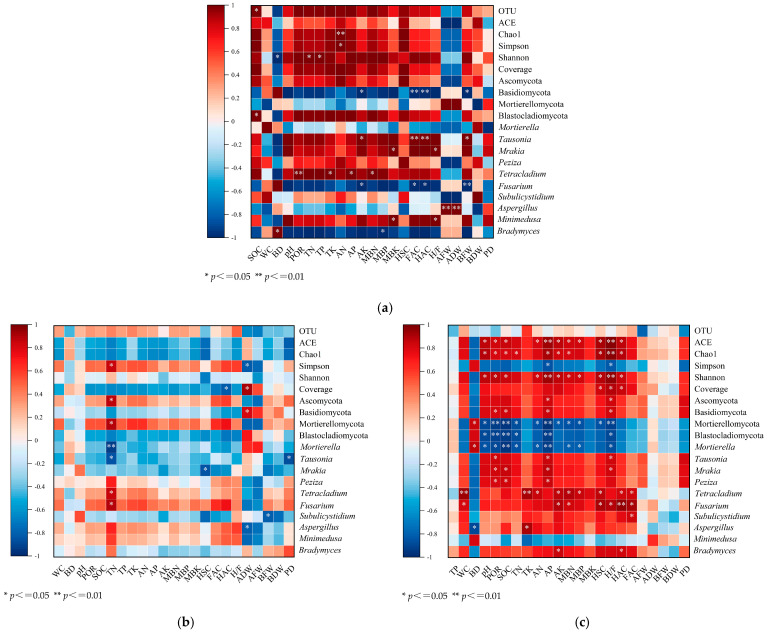
Correlation between microbial communities and soil physicochemical indicators and plant indicators: (**a**) CK treatment, (**b**) LM treatment, (**c**) CF treatment. OUT, Operational Taxonomic Unit, ACE, Abundance-based Coverage Estimator, SOC, Soil Organic Carbon, WC, Water Content, BD, Bulk Density, pH, Potential of Hydrogen, POR, Porosity, TN, Total Nitrogen, TP, Total Phosphorus, TK, Total Potassium, AN, Available Nitrogen, AP, Available Phosphorus, AK, Available Potassium, MBN, Microbial Biomass Nitrogen, MBP, Microbial Biomass Phosphorus, MBK, Microbial Biomass Potassium, HSC, Humus Carbon, FAC, Fulvic Acid Carbon, HAC, Humic Acid Carbon, H/F, Humic Acid/Fulvic Acid Ratio, AFW, Aboveground Fresh Weight, ADW, Aboveground Dry Weight, BFW, Belowground Fresh Weight, BDW, Belowground Dry Weight, PD, Pest Damage.

In the control plots, soil organic carbon (SOC) showed a strong positive association with plant biomass, which may indicate its potential role as a carbon and nutrient source that supports root uptake through the mineralization of nitrogen (N) and phosphorus (P) [73]. Bulk density (BD) exhibited a negative correlation with porosity (POR), which could constrain root penetration and water infiltration. In contrast, higher POR was positively associated with belowground dry weight (BDW), potentially due to improved aeration [74]. SOC was also positively correlated with fungal alpha diversity, as reflected by a higher Shannon index, suggesting a more balanced community structure [75]. At the taxonomic level, the abundance of Ascomycota showed a positive correlation with SOC, which might indicate its contribution to decomposition processes [76]. In contrast, Fusarium displayed low abundance, which could result from competitive suppression under conditions of higher microbial biomass (MBN/MBP) [77]. Beneficial taxa such as *Mortierella* were positively linked to plant growth, possibly through hormone production (IAA) or P solubilization, while Tetracladium appeared to contribute to organic matter breakdown [78]. Elevated Simpson index values were associated with healthier plants, suggesting that even community distribution enhances ecosystem resilience and indirectly supports growth [79]. However, higher BD (>1.7 g/cm^3^) was associated with constrained root systems and an increase in opportunistic pathogens such as Aspergillus [80] (Figure 8a).

#### 3.5.2. LM Treatment

Under litter mulching, soil physicochemical and microbial indicators demonstrated synergistic effects on plant performance. SOC was negatively correlated with Ascomycota abundance (r = −0.62 *), while Mortierellomycota exhibited a strong positive association with available phosphorus (AP, r = 0.71 **) [81]. Beneficial fungi such as *Mortierella* were enriched, enhancing nutrient mobilization and root biomass, whereas pathogenic Fusarium abundance was negatively correlated with BDW (r = −0.68 *) [82]. Increases in humic acid carbon (HSC) stimulated saprotrophic taxa like Tausonia [83], while Subulicystidium (a mycorrhizal fungus) correlated positively with aboveground biomass (r = 0.59, *p* = 0.056) [84]. Functional guilds related to phenolic metabolism, such as Minimedusa, were positively associated with fulvic acid carbon (FAC, r = 0.73 **) [85]. Overall, five years of litter mulching improved soil porosity (+18%), enhanced humus quality (HSC +15.2%), enriched beneficial fungi (+23%), and ultimately increased plant biomass by 9.8–12.3% [83]. Positive correlations between MBN and Chao1 richness indicated that higher microbial biomass promoted fungal diversity, while AP enrichment was negatively correlated with Simpson index, suggesting that excessive P availability may drive dominance of specific groups such as Ascomycota [86] (Figure 8b).

#### 3.5.3. CF Treatment

Chemical fertilization reduced fungal diversity and promoted dominance of specific taxa. Increased bulk density and reduced porosity limited hyphal growth and oxygen diffusion, thereby constraining aerobic fungi [87]. Biomass accumulation stimulated saprotrophic genera such as *Peziza*, while lignin-degrading Basidiomycota increased, raising the HA/FA ratio and enhancing organic matter turnover [88]. Stress-tolerant fungi (*Mrakia*) also expanded under nutrient imbalance [89]. Pathogen suppression was evident as Fusarium abundance correlated negatively with AN (r = −0.82 **) and MBN (r = −0.79 **) [90]. Conversely, *Mortierella* was positively linked to SOC (r = 0.74 **) and AFW (r = 0.68 **), likely via hormone precursor secretion [91]. Diversity indices confirmed strong associations between Shannon and SOC/POR, Simpson with BD (positive) and AP (negative), and Chao1 richness with TN and MBN [92]. OTU richness also correlated positively with HSC and AFW [93]. Overall, CF enhanced certain growth traits but reduced fungal stability, highlighting ecological trade-offs under intensive fertilization (Figure 8c).

### 3.6. Structural Equation Modeling of Soil–Microbe–Plant Interactions Under Different Treatments

This study provides an integrated approach by simultaneously monitoring soil physical properties, chemical properties, fungal diversity, and plant growth metrics, offering a comprehensive view of the effects of leaf mulching (LM) on agroecosystems. Unlike previous studies that typically focus on a single indicator, our research integrates these different aspects to capture the full range of impacts on soil quality and plant productivity.

**Figure 9:**
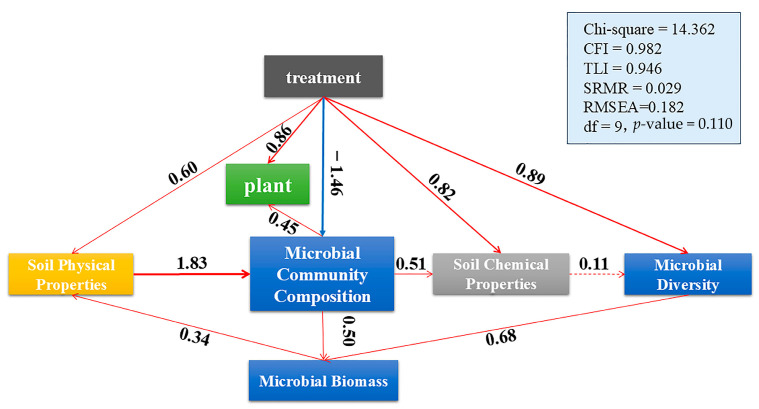
Structural equation model (SEM) illustrating the effects of different treatments on soil physical and chemical properties, microbial biomass, microbial community composition, microbial diversity, and plant growth. Standardized path coefficients are shown next to the arrows; red arrows denote significant positive relationships (*p* < 0.05). Model fit indices: χ^2^ = 14.362, df = 9, *p*-value = 0.110; CFI = 0.982; TLI = 0.946; RMSEA = 0.182; SRMR = 0.029. Solid red arrows indicate significant paths (*p* < 0.05), while blue arrows indicate weaker or non-significant relationships. Numbers represent standardized path coefficients. CFI = Comparative Fit Index; TLI = Tucker-Lewis Index; SRMR = Standardized Root Mean Square Residual; RMSEA = Root Mean Square Error of Approximation; df = Degrees of Freedom; *p*-value = probability value.

To further disentangle the direct and indirect effects of different management practices on soil health, microbial dynamics, and plant performance, we constructed a structural equation model (SEM) (Figure 9). The model exhibited a good fit to the data (χ^2^ = 14.362, df = 9, *p*-value = 0.110; CFI = 0.982; TLI = 0.946; RMSEA = 0.182; SRMR = 0.029), indicating robustness and reliability of the proposed pathways [94].

The results revealed that management treatment exerted the strongest direct influence on microbial community composition (path coefficient = 0.96) and plant biomass (0.45). Microbial community composition, in turn, played a central mediating role, linking soil properties and plant responses. Specifically, soil physical properties strongly shaped microbial communities (1.83), which subsequently regulated microbial biomass (0.59) and influenced soil chemical properties (0.51). Soil chemical properties exhibited only a weak direct effect on microbial diversity (0.11), whereas microbial community composition had a stronger effect (0.89), underscoring the pivotal role of microbial assemblages in structuring soil biodiversity [95].

Moreover, microbial biomass was positively associated with microbial diversity (0.68), suggesting that higher microbial abundance promoted functional complexity and taxonomic richness [96]. Collectively, these pathways highlight that organic mulching enhanced soil physical structure and microbial biomass, which cascaded into improved microbial diversity and plant growth. In contrast, chemical fertilization exerted weaker and less stable effects, reinforcing the ecological superiority of organic amendments [97].

Although LM supplied a higher total mass of organic material than CF, the observed benefits cannot be attributed solely to nutrient quantity. Leaves provide complex carbon sources, humic precursors, polysaccharides and lignocellulose, which enhance soil aggregation, microbial metabolism and humification processes. Multiple studies have shown that organic inputs stimulate microbial diversity and functional guilds beyond the effects of equal mineral nutrient additions [98]. Our findings therefore represent a combined effect of nutrient supply, carbon input and microbially mediated soil improvement under realistic practice conditions.

## 4. Conclusions

This study demonstrates that long-term application of decomposed leaf mulch markedly improves soil quality, microbial function, and plant performance in cold-region agroecosystems. Organic mulching reduced bulk density, enhanced porosity, and increased soil organic carbon and humus fractions, thereby creating a more favorable physical and biochemical environment for root development. These improvements supported higher microbial biomass and enriched beneficial fungal taxa (e.g., *Mortierella*), which played key roles in nutrient mobilization and plant nutrient acquisition.

High-throughput sequencing showed that leaf mulching promoted greater fungal richness, evenness, and functional diversity than conventional fertilization, which tended to reduce community stability and favor the dominance of stress-tolerant groups. Functional predictions further indicated that organic inputs better preserved saprotrophic and symbiotic functions—particularly mycorrhizal associations—whereas synthetic fertilization disrupted key trophic guilds. Structural equation modeling confirmed that shifts in microbial community composition mediated the cascading effects of mulching on soil properties and plant productivity.

At the plant level, leaf mulching produced the highest above- and belowground biomass, underscoring its potential to enhance both the yield and ecological resilience of *Aralia continentalis.* In contrast, conventional fertilization provided short-term productivity benefits but was associated with reduced microbial diversity and ecosystem stability, suggesting ecological trade-offs.

Importantly, this experiment compared two real-world management strategies rather than nutrient-equivalent inputs; thus, the observed advantages of leaf mulching likely reflect the combined effects of organic matter supply, carbon inputs, and microbial stimulation, in addition to nutrient contributions. Future studies will incorporate nutrient-standardized organic–inorganic comparisons and cost–benefit analyses to further isolate mechanistic drivers and evaluate agronomic efficiency.

Overall, our results highlight organic mulching as a scalable and ecologically sustainable practice that enhances soil health, microbial functioning, and medicinal plant productivity in black soil regions. Multi-site and multi-year studies, as well as investigations across different medicinal crops and soil types, are warranted to validate the broader applicability of this approach and explore the functional roles of key microbial taxa in agroecological systems.

## Figures and Tables

**Table 1:** Basic properties of soil under different experimental treatments.

Soil Layer (cm)	Test Treatment	pH	Moisture Content (%)	Bulk Density (g/cm^3^)	Porosity (%)
0–10	CK	6.91 ± 0.09 a	11.49 ± 1.12 c	1.56 ± 0.04 a	33.72 ± 1.50 c
	LM	5.93 ± 0.10 c	36.06 ± 0.26 a	1.07 ± 0.02 c	58.71 ± 0.29 a
	CF	6.31 ± 0.02 b	29.03 ± 0.38 b	1.33 ± 0.03 b	41.17 ± 0.27 b
10–20	CK	6.68 ± 0.15 a	10.23 ± 0.62 c	1.76 ± 0.03 a	30.84 ± 0.30 c
	LM	5.94 ± 0.05 c	31.11 ± 0.44 a	1.16 ± 0.05 c	46.84 ± 0.47 a
	CF	6.17 ± 0.08 b	26.20 ± 0.58 b	1.40 ± 0.02 b	39.69 ± 0.72 b

Note: Different letters indicate significant differences between different treatment groups of the same soil layer (*p* < 0.05).

**Table 2:** Changes in soil humus components under different experimental treatments.

Soil Layer (cm)	Test Treatment	Humus Carbon g/kg	Fulvic Acid Carbon g/kg	Humic Acid Carbo g/kg	Humic Acid/ Fulvic Acid Ratio
0–10	CK	7.14 ± 0.06 c	3.31 ± 0.19 a	1.51 ± 0.13 c	0.46 ± 0.02 c
	LM	10.53 ± 0.68 a	3. 10 ± 0.17 a	6.36 ± 0.22 a	2.06 ± 0.05 a
	CF	8.04 ± 0.24 b	2.26 ± 0.14 b	4.32 ± 0.34 b	1.91 ± 0.04 b
10–20	CK	6.68 ± 0.31 c	2.73 ± 0.15 a	1.19 ± 0.11 c	0.43 ± 0.02 c
	LM	9.51 ± 0.44 a	2.82 ± 0.11 a	5.68 ± 0.36 a	2.01 ± 0.02 a
	CF	7.06 ± 0.07 b	2.08 ± 0.04 b	3.63 ± 0.11 b	1.74 ± 0.04 b

Note: Different letters indicate significant differences between different treatment groups of the same soil layer (*p* < 0.05).

**Table 3:** Soil microbial alpha diversity indices under different treatments.

Treatment	OTU	ACE	Chao1	Simpson	Shannon	Coverage
CK	228 ± 5.2 b	242.19 ± 8.5 b	243.95 ± 7.8 b	0.07 ± 0.004 b	3.93 ± 0.05 b	0.9961
CF	430 ± 0.7 a	444.16 ± 12.9 a	447.03 ± 9.3 a	0.03 ± 0.002 a	4.38 ± 0.07 a	0.9990
LM	428 ± 9.3 a	436.99 ± 10.2 a	442.29 ± 10.0 a	0.03 ± 0.003 a	4.40 ± 0.06 a	0.9994

Note: Different letters indicate significant differences between different treatment groups of the same soil layer (*p* < 0.05).

## Data Availability

Data are contained within this article.
